# Loss of heterozygosity in the Hodgkin-Reed Sternberg cell line L1236

**DOI:** 10.1054/bjoc.2000.1593

**Published:** 2001-02

**Authors:** A Staratschek-Jox, R Kurt Thomas, T Zander, N Massoudi, M Kornacker, J Bullerdiek, C Fonatsch, V Diehl, J Wolf

**Affiliations:** Department of Internal Medicine I, University of Cologne, Joseph Stelzmann Str. 9, Cologne, 50931, Germany; Center of Human Genetics and Genetic Counselling, University of Bremen, Leobener Str. ZHG, Bremen, 28359, Germany; Institut für Medizinische Biologie der Universität Wien, Währinger Str. 10, Wien, 1090, Austria

**Keywords:** Hodgkin's disease, loss of heterozygosity, tumour suppressor gene

## Abstract

Hodgkin-Reed Sternberg cells are derived from germinal centre B-cells in most cases. Somatic mutations affecting their rearranged immunoglobulin genes were detected, rendering potential functional rearrangements non-functional. Under physiological conditions such cells would be designated to undergo apoptosis within the germinal centre. In search for the specific transforming event that prevents Hodgkin-Reed Sternberg cells from programmed cell death, cytogenetic analyses were broadly performed but did not reveal specific chromosomal aberrations. Analysis of these cells on the molecular level is difficult to perform due to the scarcity of the cells in the lymphoma tissue and the given limitations of in situ studies. To overcome these limitations, the cell line L1236, known to be derived from Hodgkin-Reed Sternberg cells in situ, was chosen for allelotype analysis. Using a panel of microsatellite loci assigned to nearly all chromosomal arms, regions of loss of heterozygosity were detected on chromosomal arms 6p, 9q and 17p. The size of lost segments was estimated by amplification of additional microsatellite loci mapped to the respective regions. Further analyses of single Hodgkin-Reed Sternberg cells will reveal whether LOH affecting these regions is a recurrent event in HD and to which extent the smallest commonly affected region can be estimated. © 2001 Cancer Research Campaign http://www.bjcancer.com


*British Journal of Cancer* (2001) **84**(3), 381–387
© 2001 Cancer Research Campaign
doi: 10.1054/ bjoc.2000.1593, available online at http://www.idealibrary.com on IDEAL®

### Loss of heterozygosity in the Hodgkin-Reed Sternberg cell line L1236

**A Staratschek-Jox[1], R Kurt Thomas[1], T Zander[1], N Massoudi[1], M Kornacker[1], J Bullerdiek[2], C Fonatsch[3], V Diehl[1] and J Wolf[1]**

[1]Department of Internal Medicine I, University of Cologne, Joseph Stelzmann Str. 9, 50931 Cologne, Germany; [2]Center of Human Genetics and Genetic Counselling, University of Bremen, Leobener Str. ZHG, 28359 Bremen, Germany; [3]Institut für Medizinische Biologie der Universität Wien, Währinger Str. 10, 1090 Wien, Austria

**Summary** Hodgkin-Reed Sternberg cells are derived from germinal centre B-cells in most cases. Somatic mutations affecting their rearranged immunoglobulin genes were detected, rendering potential functional rearrangements non-functional. Under physiological conditions such cells would be designated to undergo apoptosis within the germinal centre. In search for the specific transforming event that prevents Hodgkin-Reed Sternberg cells from programmed cell death, cytogenetic analyses were broadly performed but did not reveal specific chromosomal aberrations. Analysis of these cells on the molecular level is difficult to perform due to the scarcity of the cells in the lymphoma tissue and the given limitations of in situ studies. To overcome these limitations, the cell line L1236, known to be derived from Hodgkin-Reed Sternberg cells in situ, was chosen for allelotype analysis. Using a panel of microsatellite loci assigned to nearly all chromosomal arms, regions of loss of heterozygosity were detected on chromosomal arms 6p, 9q and 17p. The size of lost segments was estimated by amplification of additional microsatellite loci mapped to the respective regions. Further analyses of single Hodgkin-Reed Sternberg cells will reveal whether LOH affecting these regions is a recurrent event in HD and to which extent the smallest commonly affected region can be estimated. © 2001 Cancer Research Campaign http://www.bjcancer.com

**Keywords**: Hodgkin's disease; loss of heterozygosity; tumour suppressor gene

Hodgkin-Reed Sternberg (H-RS) cells, which are the malignant cells in Hodgkin's disease (HD), clonally derive from germinal centre B-cells. The occurrence of somatic mutations rendering potential functional immunoglobulin (Ig) gene rearrangements non-functional were described as a characteristic feature of H-RS cells, leading to loss of expression of a high affinity antibody in these cells (Küppers and Rajewsky, 1998). Although under physiological conditions those cells would be committed to undergo programmed cell death, H-RS cells do escape apoptosis. Thus, the mechanism leading to apoptosis resistance of H-RS cells should be further elucidated since it plays a crucial role in the development of Hodgkin's lymphoma.

Recently, evidence for the implication of IκBα as a tumour suppressor gene was obtained in some cases of HD. In 2 out of 8 HD-derived cell lines deleterious mutations were detected in one allele while the other allele was lost. Moreover, fatal clonal mutations affecting the IκBα gene were also observed in H-RS cells obtained from 2 out of 5 HD biopsy specimen, most probably leading to the expression of a truncated IκBα protein (Jungnickel et al, 2000). Since IκBα downregulates NFκB, loss of function of IκBα would lead to constitutive activation of NFκB mediating anti-apoptotic signals. Indeed constitutive activation of NFκB in H-RS cells can be detected in a substantial proportion of HD cases (Bargou et al, 1997). On the other hand, as was already mentioned, cases of HD in which the H-RS cells express wild type IκBα exist. For example in L1236 cell line, which is known to be clonally derived from H-RS cells, presence and transcription of two IκBα wild type alleles was demonstrated making an alteration of IκBα leading to the transformation of these lymphoma cells very unlikely. Thus, the identification of clonal genetic alterations for which H-RS cells were selected for in these cases, is needed.

Since IκBα was already identified to be involved in HD pathogenesis as a tumour suppressor gene, further studies should address the identification of loss of function of other genes in HD. Recently, Hasse et al (1999) provided evidence for the presence of loss of heterozygosity (LOH) in H-RS cells. Four different loci were investigated by amplification for microsatellite DNA. In 6 out of 7 cases LOH was detected affecting at least one of these loci. Regions of LOH occurring in H-RS cells may refer to the presence of tumour suppressor genes that are involved in the pathogenesis of Hodgkin's lymphoma. It would thus be of interest to further characterize the pattern of LOH by analysing additional microsatellite loci covering nearly each arm of every chromosome. However, since HD derived lymphoma tissues consist only of a small amount of H-RS cells (up to 5%) comprehensive allelotyping of tumour cells cannot be performed easily because of the need of a 'pure' lymphoma cell population. Efforts to enrich primary H-RS cells from lymph node suspensions were successful but only a small number of cells were recovered and still a substantial proportion of other cells were admixed (Irsch et al, 1997). Established HD derived cell lines which are supposed to be of H-RS cell origin and thus most probably represent the lymphoma cell population could not be used for allelotype analysis since the germ line allelotype of the patient could not be determined due to the lack of normal control tissues of the respective patient.

*Received 2 May 2000*
*Revised 14 October 2000*
*Accepted 17 October 2000*

*Correspondence to:* A Staratschek-Jox

The recently established EBV-negative cell line L1236 was shown to be derived from the patient's clonal H-RS cell population by amplifying identical Ig gene rearrangements from the cell line as well as from H-RS cells micromanipulated from fresh frozen sections of the patient's bone marrow (Kanzler et al, 1996). Thus, L1236 represents a valid population of H-RS cells. From the same patient a bone marrow sample was obtained during complete clinical remission that could be analysed to define the germ line allelotype of the patient. In the present study allelotype analyses using a comprehensive panel of oligonucleotides in order to amplify microsatellite DNAs located on each arm of each chromosome were performed to detect regions of clonal loss of heterozygosity in L1236 cells that may have implications for the pathogenesis of HD.

### MATERIALS AND METHODS

### Cell line L1236

L1236 is an EBV-negative cell line which was established from the peripheral blood of a patient suffering from relapse of HD of mixed cellularity subtype (Wolf et al, 1996). Its origin from primary H-RS cells was proven by amplification of identical Ig gene rearrangements from the cell line as well as from H-RS cells micromanipulated from the patients bone marrow during relapse (Kanzler et al, 1996). The cell line IARC 277 is an EBV immortalized lymphoblastoid cell line and was kindly provided by G Lenoir (Lenoir et al, 1985). This cell line was used as a control population to determine the sensitivity of detection of L1236 cells within a population of non-malignant B-cells.

### Bone marrow cells

The bone marrow cells (BMC) of the same patient whose peripheral blood cells were used to establish the cell line L1236, were harvested for autologous bone marrow transplantation during complete clinical remission (for case report of the patient see Jox et al (1998) and used as normal tissue control for determination of the patient's germ line allelotype.

### Oligonucleotides

According to Osborn and Leech (1994) oligonucleotides were chosen for amplification of microsatellite DNA representing each arm of every chromosome except for the short arms of the acrocentric chromosomes 13, 14, 15, 21 and 22 (see Table 1). To map the extent of detected loci with LOH neighbouring microsatellite loci obtained from the Genethon linkage map (http://www.genethon.fr/genethon_en.html) and the NCBI data base (http://www.ncbi.nlm.nih.gov/) were further analysed (for sequence information of oligonucleotides and references of microsatellite loci see Table 2). For amplification of the H-RS cell specific $V_H1$ Ig gene rearrangement oligonucleotides were chosen from the leader region (VH1L: 5′-′TCACCATGGACTGGACCTGGAG-3′), from the FR1 region (F10VH1: 5′-GCCTGGGGCCTCAGTGAAGGT-3′) as well as from the CDR3 region (3H1: 5′-GTGGCCATTGTAGTTCCCAC-3′).

### DNA extraction

For amplification of microsatellite loci high molecular weight DNA was extracted from L1236 cells and the patient's bone marrow using standard protocols (Sambrock et al, 1989). PCR sensitivity for amplification of the H-RS cell specific $V_H1$ Ig gene rearrangement from DNA obtained from the patient's bone marrow was estimated by mixing L1236 cells to IARC 277 cells in various concentrations.

### PCR analysis

PCR analyses were performed in a reaction volume of 50µ containing 50 mM KCl, 2.5 mM $MgCl_2$, 200 µM dNTP, 25 µM of each primer. 1 unit of Taq DNA polymerase (Promega, Mannheim, Germany) was added during the first denaturation step. For detection of LOH 5 ng template DNA was used. For all reactions PCR consisted of 60 s at 95˚C, 120 s at different annealing temperatures given in Table 1 and Table 2 and 120 s at 72˚C for 35 cycles, followed by a final extension for 7 min at 72˚C. For detection of the $V_H1$ gene rearrangement PCR consisted of two rounds of PCR at 90 s at 95˚C, 30 s at 57˚C and 80 s at 72˚C for 40 cycles, followed by a final extension for 7 min at 72˚C. PCR products were separated on an agarose gel and visualized by ethidium bromide staining or on a denaturing polyacrylamide gel followed by silver staining, respectively.

### Southern blot analysis

PCR products were separated by agarose gel electrophoresis and transferred onto a nylon filter (Gene Screen Plus; NEN, Boston, USA) using standard methods (Sambrock et al, 1989). To detect the H-RS cell specific $V_H1$ gene rearrangement an internal oligonucleotide (5-1 CDR2: 5′-CCGGTGTTGGCTCGACAATG-3′) was labelled and hybridized using the ECL kit (Roche Biochemicals, Mannheim, Germany) according to the manufacturer's protocol.

### Silver staining of DNA separated on polyacrylamide gels

PCR products were separated in a 5% polyacrylamide gel (Electrophoresis apparatus Model S2, Renner, Eggenstein, Germany). For fixation of DNA the gel was incubated in 10% glacial acetic acid (Baker Chemikalien, Gross Gerau, Germany) for 10 min and then rinsed three times in distilled water. Thereafter the gel was impregnated in an AgNO3 solution (Merck, Darmstadt, Germany) (1 g $l^{-1}$) containing 0.05% formaldehyde (Baker Chemikalien, Gross Gerau, Germany) for 30 min. After decanting the $AgNO_3$ solution the gel was rinsed for a few seconds in distilled water. For reduction of $AgNO_3$ the gel was incubated in a solution consisting of $NaCO_3$ (30 g $l^{-1}$) (Merck, Darmstadt, Germany), sodium thiosulphate (2 mg $l^{-1}$) (Merck, Darmstadt, Germany) and formaldehyde (0.05%). The reduction process was stopped with 10% glacial acetic acid.

### RESULTS

### Analysis of the patient's bone marrow cells for minimal residual disease

For allelotype analysis of lymphoma cells a nearly pure population of non-malignant cells obtained from the same patient is needed to determine the patient's germ line allelotype. Therefore, the patient's bone marrow cells (BMC), which were obtained

 *© 2001 Cancer Research Campaign*

during complete clinical remission, were analysed to exclude minimal residual disease. The $V_H1$ gene rearrangement of L1236 cells was already used to detect the patient's H-RS cells in various tissues obtained during the course of the disease (Jox et al, 1998). To test the patient's BMC for the presence of H-RS cells a semi-nested PCR was performed. Simultaneously, L1236 cells were mixed with B-lymphoblastoid cells (IARC 277) in various concentrations to estimate the sensitivity of the PCR approach. Approximately 5 ng DNA obtained from the patient's bone marrow cells as well as from the mixture of cell lines were used as template in the first PCR round. In the second round 1 μl of the first round was utilized. Given the sensitivity of the detection of one L1236 cell within $10^4$ 1ARC 277 cells in subsequent Southern blot analysis of the PCR products, no H-RS cells were detected in the BMC sample. Thus, BMC cells do not contain H-RS cells in a proportion that could interfer analysis of the patient's allelotype. For allelotype analysis 5 ng of BMC DNA was subsequently use as control.

### Allelotyping of L1236 cells revealed LOH on three chromosomal loci

Using a comprehensive set of 112 oligonucleotides (Osborn and Leech, 1994) to amplify at least one microsatellite locus on each chromosomal arm except for the short arms of acrocentric chromosomes, the amplification of most of the microsatellite loci revealed no LOH. Of the total number of 56 amplified microsatellites, 43 were informative revealing the detection of heterozygosity in the patient's germ line allelotype. On three chromosomal loci i.e. 6p (FTHP1), 9q(D9s15), and 17p (L02016) LOH was detected (Table 1).

### Mapping the extent of LOH on the affected chromosomal loci

To map the extent of LOH on each chromosomal area that was detected to be involved by allelic loss, additional microsatellite loci, which are located in the proximity of the initial locus, were identified for amplification (Table 2). On chromosome 6p, 12 repeats were further amplified. Two regions of LOH were defined, mapping to the chromosomal region 6p21-22 (see Table 2 and Figure 1). Each region comprises about 4–6 megabases whereby in the proximal region retention of an area of heterozygosity occurs (Figure 1A and Table 2A). Similarly the extent of LOH on 9q was mapped by amplifying 15 additional microsatellite loci. The proximal breakpoint was found to be located between D9s1777 and D9s1876 while the distal breakpoint is located between D9s1812 and D9s278. Thus, the region of LOH comprises about 36 megabases (Figure 1B and Table 2B). For further analysis of LOH on 17p, 8 additional loci were amplified. 5 loci chosen from 17p revealed allelic loss allowing to map the breakpoint between D17s926 and D17s805. Within this region amplification of one microsatellite locus revealed retention of heterozygosity. Thus, allelic loss on chromosome 17 extends to nearly the whole short arm and comprises about 27 megabases whereby a region of heterozygosity was retained (Figure 1C and Table 2C).

### DISCUSSION

The detection of specific chromosomal aberrations in HD may be hampered by the small amount of H-RS cells in the primary tissue and their low mitotic index compared to the surrounding non-malignant lymphocytes. Conventional cytogenetic analysis of the

**Table 1** Allelotyping of L1236 cells

| Chromosome (band) | Locus | Result (L1236) | Chromosome (band) | Locus | Result (L1236) |
|---|---|---|---|---|---|
| 1p21 | AMY2B | heterozygous | 9q34.1 | ASS | not informative |
| 1q21-23 | APOA2 | not informative | 10p11,2-pter | D10S89 | heterozygous |
| 1q32-q44 | DIS103 | heterozygous | 10p11.2-pter | D10S111 | heterozygous |
| 2p23-pter | TPO | heterozygous | 10p | D10S179 | not informative |
| 2p12 | CD8A | not informative | 11p13-15.1 | D11S419 | heterozygous |
| 2q33-35 | D2S72 | not informative | 11q13 | D11S534 | heterozygous |
| 3q21-qter | ACPP | not informative | 11q23.2-24 | D11S836 | not informative |
| 4p11-15 | D4S174 | heterozygous | 12p12-pter | F8VWF | heterozygous |
| 4p12-13 | GABARBI | heterozygous | 12q22-q24.33 | D12S60 | heterozygous |
| 4q25-34 | D4175 | heterozygous | 13q12 | FLT-1 | heterozygous |
| 4q35-qter | D4S171 | not informative | 13q11-12.1 | D13S115 | heterozygous |
| 5p15 | D5S268 | heterozygous | 14q31 | D14S34 | heterozygous |
| 5p15.1-15.3 | D5S117 | heterozygous | 15q26.1 | FES | not informative |
| 5q21-22 | D5S346 | heterozygous | 16p13 | D16S292 | heterozygous |
| 6p24-25 | F13A1 | heterozygous | 16q21 | D16S265 | heterozygous |
| 6p21.3-p24 | D6S109 | not informative | 17p13.1 | TP53 | LOH |
| 6p12-21.3 | FTHP1 | LOH | 17q11.2-12 | D17S250 | heterozygous |
| 6q23.1 | D6S87 | heterozygous | 17q12-21 | D17S588 | heterozygous |
| 7p11.2-12 | EGFR | heterozygous | 18p11.21-pter | D18S40 | heterozygous |
| 7q31 | D7S23 | heterozygous | 18q21.2-21.3 | D18S35 | not informative |
| 7q31 | CFTR | heterozygous | 19q12 | D19S49 | heterozygous |
| 8p22 | LPL | heterozygous | 20p12 | D20S27 | heterozygous |
| 8p11.2-11.1 | D8S135 | heterozygous | 20q12 | D20S46 | heterozygous |
| 8p11.1-21.1 | ANKI | heterozygous | 21p11.2 | D21S120 | not informative |
| 8q22-qter | D8S161 | heterozygous | 21q22.3-qter | D21S171 | heterozygous |
| 9p22-pter | D9S54 | heterozygous | 22q | D22S156 | heterozygous |
| 9q13-21 | D9S15 | LOH | 22q11.2-13.1 | TOPIP2 | heterozygous |
| 9q33 | GSN | heterozygous | Xq21.1-23 | DX454 | not informative |

Amplification of 56 microsatellite loci revealed the detection of LOH at three chromosomal loci i.e. 6p12-21.3, 9q13-21 and 17p13.1. The microsatellite loci and oligonucleotides were chosen according to Osborn and Leech (1994)

© 2001 Cancer Research Campaign 

**Table 2 A–C** Mapping the extent of LOH on three chromosomal loci

| Chromosome (band) | Comp | Locus | Accession | Oligonucleotides (5'-3') | A-Temp. | Result (L1236) |
|---|---|---|---|---|---|---|
| **A** | | | | | | |
| 6p22 | 28.252 | D6s276 | Z16711 | atcatccccagaaggaacact | | |
| | | | | gtgcaacttgttcctcctttg | 57 ˚C | LOH |
| 6p22.3-p21.3 | 32.300 | D6s306 | Z17120 | agaattgaccttccaattcacc | | |
| | | | | caaatttgaagtggtggggt | 53 ˚C | heterozygous |
| 6p21.3 | 36.035 | D6s439 | Z23903 | aactcaggcttcacaactttgg | | |
| | | | | agcctcagggaagacacatttt | 59 ˚C | LOH |
| 6p21.3 | 36.855 | D6s1629 | Z53381 | gaacgtactgtcccacacactt | | |
| | | | | tgatgctagggaaggggcatct | 61 ˚C | LOH |
| 6p21.3 | 38.016 | D6s1576 | Z52627 | aaaactattgggggagatgga | | |
| | | | | ggcctgtgtaagtgcattcta | 56 ˚C | heterozygous |
| 6p21.3 | 38.987 | D6s1610 | Z53131 | ccacttctcctggtgagataga | | |
| | | | | gttaggaatttccagcagagcc | 61 ˚C | heterozygous |
| 6p21.2-p21.1 | 40.531 | D6s1562 | Z51063 | agtgcaggtccttcttcatacc | | |
| | | | | tggaactaagctttacagcccc | 61 ˚C | LOH |
| 6p21.2-p21. | 42.192 | D6s426 | Z23755 | tctgttgagttctccctctccc | | |
| | | | | tggaaccagaaactgtggcata | 57 ˚C | LOH |
| 6p21.2-p21.1 | 44.305 | D6s1632 | Z53424 | caccagtactatgccaggcata | | |
| | | | | tgagggctctggttcttattgc | 61 ˚C | LOH |
| 6p21.2-p21.1 | 44.396 | D6S1566 | Z52556 | aaatatacctcttcactggcc | | |
| | | | | cagcattatgttctcctgtct | 57 ˚C | LOH |
| 6p12 | 44.442 | D6S452 | Z24209 | ggatctaaatctcacccgtac | | |
| | | | | aagttctgggtgattcaggt | 55 ˚C | LOH |
| 6p21.2-p21.1 | 44.688 | D6s438 | Z23901 | taagtgaggggaacatcacaga | | |
| | | | | acatgtgcagaatgagcagatt | 62 ˚C | heterozygous |
| **B** | | | | | | |
| 9p12-q11 | 55.403 | D9s1777 | Z52124 | gtaccaaggcaatgctggttca | | |
| | | | | agctcccaaatacctgttaccc | 61 ˚C | heterozygous |
| 9q13-q21.1 | 58.237 | D9s1806 | Z52772 | gaggcagaggttgcagttagaa | | |
| | | | | tggcaagtagaagggagcaaca | 61 ˚C | not informative |
| 9q13-q21.1 | 59.182 | D9s1876 | Z51715 | gatgtacccagagaagtctcg | | |
| | | | | gatctatttctactcactgccag | 57 ˚C | LOH |
| 9q13-q21.1 | 60.032 | D9s1837 | Z53339 | accaaactgttcatgatggtgg | | |
| | | | | tttttcttttccctctgcccca | 56 ˚C | LOH |
| 9q13-q21.1 | 61.086 | D9s1822 | Z53100 | gtttggcttctgctgtaagggt | | |
| | | | | ctgagtgtttcaggaagcacca | 61 ˚C | LOH |
| 9q12-q21.1 | 70.836 | D9s284 | Z24432 | acttacccacatcatctgtgag | | |
| | | | | accacataaaaactctctgcc | 55 ˚C | not informative |
| 9q21.1 | 73.167 | D9s175 | Z17021 | gatagcagtaagttcttcctgg | | |
| | | | | cctcacaaactcttatcagcc | 59 ˚C | LOH |
| 9q21.3-q22 | 85.550 | D9s1807 | Z52789 | tctcccaaagttgaagagcatt | | |
| | | | | ttagtctgtggcacaagtcag | 55 ˚C | LOH |
| 9q21.1-q22 | 85.765 | D9s1834 | Z53271 | aagagagagagacagacacac | | |
| | | | | ggttaagtaactggtggagga | 57 ˚C | LOH |
| 9q21.3-q22 | 86.971 | D9s1860 | Z53953 | tgtccccaagactttcttaagg | | |
| | | | | gatcatgaagtgggcataatgc | 56 ˚C | not informative |
| 9q13-q21.1 | 91.419 | D9s153 | Z16442 | cagcccaaatggactaagaca | | |
| | | | | cagttctgaggccatatgtct | 57 ˚C | not informative |
| 9q21-q22.1 | 92.864 | D9s1785 | Z52241 | gtactgggcaatatgcaaagg | | |
| | | | | taatggaggtgcagagtacac | 57 ˚C | LOH |
| 9q21-q22.1 | 93.281 | D9s1877 | Z51753 | taagataggggctttgggctca | | |
| | | | | tgccactgggtcttaacgtgta | 61 ˚C | not informative |
| 9q22.1 | 95.348 | D9s1812 | Z52886 | ccatgactgcttcacccaacaa | | |
| | | | | tgtcccctaactgctttggatg | 61 ˚C | LOH |
| 9q22.1-q22.2 | 96.840 | D9s278 | Z24157 | gaatgttcccaggcactgata | | |
| | | | | agggtttttggagacacaga | 56 ˚C | heterozygous |

 *© 2001 Cancer Research Campaign*

**C**

| | | | | | | |
|---|---|---|---|---|---|---|
| 17p13 | 2.789 | D17s926 | Z23575 | ggctgaagtgggaagattgctt | | |
| | | | | tgaagttccgcagaaggctgtt | 61 ˚C | LOH |
| 17p13.3 | 6.033 | D17s1828 | Z53084 | ccacaggtgcactcacagattt | | |
| | | | | cctggattcagccatacctgaa | 61 ˚C | heterozygous |
| 17p13.3 | 6.298 | D17s1876 | Z51792 | gctgcttctgcaaagatgac | | |
| | | | | gttgagattacagacgtgagc | 61 ˚C | LOH |
| 17p13.1 | 11.215 | D17s1791 | Z52229 | cctgaagatgtttctggagcag | | |
| | | | | acatttgtgggtgggtggagtt | 61 ˚C | LOH |
| 17p11.2 | 16.066 | D17s1856 | Z53861 | gcatggtggtaggcacctatat | | |
| | | | | tagcagcagtccccaacctttt | 61 ˚C | LOH |
| 17p11.2-p11.1 | 27.910 | D17s805 | Z17037 | ggctgagattgcaccattgc | | |
| | | | | acagaggaccatgtgtacac | 61 ˚C | LOH |
| 17p11.1 | 27.947 | D17s1871 | Z51496 | gcacgtggcctattatgagact | | |
| | | | | ggtagggttctccagagaaaca | 61 ˚C | heterozygous |
| 17q12 | 37.973 | D17s927 | Z23601 | cctcctccaaggaaagaactga | | |
| | | | | tgacattggaagtctgacctgg | 61 ˚C | heterozygous |

By amplification of additional microsatellite loci, the extent of LOH was estimated on the three loci for which LOH was detected by allelotype analysis. The location of the microsatellite on the chromosome is given as referred to the Genatlas database by Jean Frezal (http://bisance.citi2.fr/GENATLAS/link_an.html) and to The Genetic Location Data Base, Department of Human Genetics, University of Southampton, Genetic Epidemiology Research Group (http://cedar.genetics.soton.ac.uk/public_html/ldb.html). Comp: composite location ordered by the distance of the locus from the p-telomere of the chromosome in megabases, A-temp: annealing temperature.

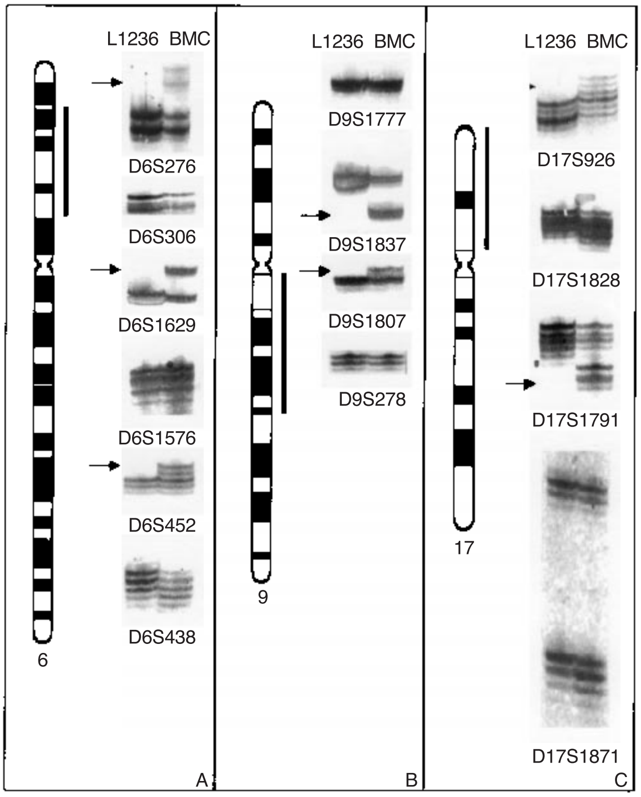

**Figure 1** **A**–**C** Loss of heterozygosity in L1236 cells. Allelotype analysis of L1236 cells was performed by amplification of microsatellite loci. Three regions of LOH were detected on 6p, 9q and 17p and analysed in detail. The ideograms of the respective chromosomes are given and the analysed regions are indicated by black vertical lanes. Examples of PCR results are given on the right of each ideogram ordered by the distance of the locus from the p-telomere of the chromosome. Examples of LOH are marked by arrows. Further information on the location of each microsatellite locus is given in Table 2. BMC: bone marrow cells newly established H-RS cell line L1236 revealed a grossly aberrant karyotype (Wolf et al, 1996; Fonatsch et al, 1999). The number of chromosomes varies from metaphase to metaphase. A similar observation was made for H-RS cells obtained from freshly prepared single cell suspensions of biopsy materials using interphase FISH in combination with immunophenotype analysis (Weber-Matthiesen et al, 1995) indicating an intrinsic chromosomal instability of these cells as already suggested by others (Fonatsch et al, 1982; Palzetti et al, 1999). Thus, conventional cytogenetic analyses as well as interphase FISH may be of limited value to define clonal aberrations present in each H-RS cell.

The molecular allelotype analysis of tumour cells enables the detection of clonal loss of heterozygosity by the amplification of microsatellite DNA using PCR and may thus offer an advantage compared to cytogenetic analysis. So far, allelotyping of H-RS cells representing every arm of nearly all chromosomes was not performed because of the need of a pure lymphoma cell population and the difficulties to enrich H-RS cells from lymph node suspensions et al, 1997). In addition, the allelotype of HD-derived cell lines could not be determined since autologous non-affected tissue samples were not available for comparison. For the H-RS cell line L1236, bone marrow cells of the respective patient obtained during clinical complete remission were available, that were screened for the absence of H-RS cells using clone specific oligonucleotides to amplify the H-RS cell specific IgH gene rearrangement. Thus, the H-RS cell line L1236 together with the patient's normal bone marrow cells for the first time enabled comprehensive allelotype analyses of H-RS cells.

Allelotype analyses revealed detection of LOH on 6p, 9q and 17p. The extent of LOH on each chromosome was estimated by amplification of adjacent microsatellite loci and was found to range between 4 and 36 megabases. Especially on chromosome 17 nearly the whole short arm of one chromosome was lost. Since the corresponding chromosomal regions were not involved in

© 2001 Cancer Research Campaign chro mosomal translocations and did not show microscopically visible deletions (Wolf et al, 1996; Fonatsch et al, 1999). LOH most probably resulted from mitotic recombination events. The retention of heterozygosity within regions of LOH further supports this view indicating that several recombination events have led to allelic loss. The detection of LOH using PCR indicates that these alterations represent clonal events that are present in most if not all L1236 cells. However, since L1236 cells grow in culture, it can not be thoroughly excluded that LOH in L1236 is caused by genetic instability in vitro and reflects the presence of various subpopulations expanding during culturing.

LOH affecting a chromosomal region in a tumour cell often points to the presence of a tumour suppressor gene that is involved in the pathogenesis of the tumour. The chromosomal regions affected by LOH in L1236 cells are too large to allow for the identification of a candidate tumour suppressor gene involved in HD. However, occurrence of LOH comprising these regions were described for other tumour entities, supporting the view that loss of a particular gene within these areas can contribute to malignant transformation. The well characterized tumour suppressor gene p53 maps to 17p. Analysis of exons 5 to 10 of the remaining p53 allele in L1236 cells revealed wild-type sequence (data not shown) further supporting the view that p53 is usually not altered by somatic mutation in H-RS cells (Montesinos-Rongen et al, 1999). Frequent LOH on 17p independent of p53 has also been observed for a variety of solid tumours (Chattopadhyay et al, 1997; Grebe et al, 1997; Steichen-Gersdorf et al, 1997; Konishi et al, 1998; Liscia et al, 1999) further providing evidence that other tumour suppressor genes may map to 17p. The region on 6p that is affected by LOH in L1236 cells covers the HLA locus. HLA-haplotype loss may confer a selective advantage for tumour cells and can be observed in a variety of neoplasias (Jimnez et al, 1999). In addition, allelic loss of the tumour necrosis factor alpha gene mapped to 6p21.3 was observed for colorectal cancer cells (Honchel et al, 1996) probably contributing to the maintenance of the malignant phenotype. Moreover, frequent allelic loss of a chromosomal fragment mapping to 6p21 was detected in diffuse large B-cell non Hodgkin's lymphoma (Nagai et al, 1999) further suggesting the existence of a putative tumour suppressor gene within this region. The presence of a tumour suppressor gene is also suggested for the chromosomal region 9q13-q21.2 since allelic loss of this region was frequently observed in carcinoma of the bladder (Keen and Knowles, 1994).

The relevance of allelic loss affecting the respective regions for the development of HD cannot be estimated by analysis of a single case. However, screening of L1236 cells for LOH enabled us to identify single regions of interest which can now further be analysed using single cell PCR on primary H-RS cells obtained from lymph node suspensions of a large number of HD cases. These analyses will reveal if LOH affecting 6p, 9q, or 17p is a frequent event in HD.

### ACKNOWLEDGEMENT

This work was supported by the Deutsche Forschungsgemeinschaft through SFB502.

### REFERENCES

Bargou RC, Emmerich F, Krappmann D, Bommert K, Mapara MY, Arnold W, Royer HD, Grinstein E, Greiner A, Scheidereit C and Dörken B (1997) Constitutive nuclear factor-kappaB-RelA activation is required for proliferation and survival of Hodgkin's disease tumour cells. *J Clin Invest* **100**: 2961–2969

Chattopadhyay P, Rathore A, Mathur M, Sarkar C, Mahapatra AK and Sinha S (1997) Loss of heterozygosity of a locus on 17p13.3, independent of p53, is associated with higher grades of astrocytic tumours. *Oncogene* **15**: 871–874

Fonatsch C, Schaadt M, Kirchner HH and Diehl V (1982) Sister chromatid exchange in lymphoma cell lines and lymphoblastoid cell lines. In: *Sister-chromatid-exchange test*, Müller D, Natarajan AT, Obe G, Röhrborn G (eds.). pp 51–64, Thieme Stuttgart: New York

Fonatsch C, Jox A, Streubel B, Wolf J and Diehl V (1999) Cytogenetic findings in Hodgkin's disease. In: *Hodgkin's disease*, P. Mauch, J. Armitage, V. Diehl, R. T. Hoppe, L. Weiss (eds), pp 213–219. Lippincott Raven: USA

Grebe SK, McIver B, Hay ID, Wu PS, Maciel LM, Drabkin HA, Goellner JR, Grant CS, Jenkins RB and Eberhardt NL (1997) Frequent loss of heterozygosity on chromosomes 3p and 17p without VHL or p53 mutations suggests involvement of unidentified tumour suppressor genes in follicular thyroid carcinoma. J *Clin Endocrinol Metab* 82: 3684–3691

Hasse U, Tinguely M, Leibundgut EO, Cajot JF, Garvin AM, Tobler A, Borisch B and Fey MF (1999) Clonal loss of heterozygosity in microdissected Hodgkin and Reed-Sternberg cells. *J Natl Cancer Inst* **91**: 1581–1583

Honchel R, McDonnell S, Schaid DJ and Thibodeau SN (1996) Tumour necrosis factor-alpha allelic frequency and chromosome 6 allelic imbalance in patients with colorectal cancer. *Cancer Res* **56**: 145–149

Jimnez P, Cantn J, Collado A, Cabrera T, Serrano A, Real LM, Garcia A, Ruiz-Cabello F and Garrido F (1999) Chromosome loss is the most frequent mechanism contributing to HLA haplotype loss in human tumors. *Int J Cancer* **83**: 91–97

Jox A, Zander T, Kornacker M, Kanzler, H, Küppers R, Diehl V and Wolf J (1998) Detection of identical Hodgkin-Reed Sternberg cell specific Immunoglobulin Gene rearrangement in a patient with Hodgkin's disease of mixed cellularity subtype at primary diagnosis and in relapse two and a half years later. *Ann Oncol* **9**: 283–287

Jungnickel B, Staratschek-Jox A, Brauninger A, Spieker T, Wolf J, Diehl V, Hansmann ML, Rajewsky K and R. Küppers R (2000) Clonal deleterious mutations in the I{kappa}B{alpha} gene in the malignant cells in Hodgkin's lymphoma. *J Exp Med* **191**: 395–402

Kanzler H, Hansmann M-L, Kapp U, Wolf J, Diehl V, Rajewsky K and Küppers R (1996) Molecular single cell analysis demonstrates the derivation of a peripheral blood derived cell line (L1236) from the Hodgkin/Reed-Sternberg cells of a Hodgkin's lymphoma patient. *Blood* **87**: 3429–3426

Keen AJ and Knowles MA (1994) Definition of two regions of deletion on chromosome 9 in carcinoma of the bladder. *Oncogene* **9**: 2083–2088

Konishi H, Takahashi T, Kozaki K, Yatabe Y, Mitsudomi T, Fujii Y, Sugiura T, Matsuda H, Takahashi T and Takahashi T (1998) Detailed deletion mapping suggests the involvement of a tumour suppressor gene at 17p13.3, distal to p53, in the pathogenesis of lung cancers. *Oncogene* **17**: 2095–2100

Küppers R and Rajewsky K (1998) The origin of Hodgkin and Reed Sternberg cells in Hodgkin's disease. *Annu Rev Immunol* **16**: 471–493

Lenoir GM, Vuilaume M and Bonnardel C (1985) The use of lymphomatous and lymphoblastoid cell lines in the study of Burkitt's lymphoma. In: *Burkitt's lymphoma: A human cancer model*. G. M. Lenoir, G. T. O'Conor and C. L. M. Olweny (eds.), pp. 309–318. IARC Scientific Publications, No. 60, IARC, Lyon

Liscia DS, Morizio R, Venesio T, Palenzona C, Donadio M and Callahan R (1999) Prognostic significance of loss of heterozygosity at loci on chromosome 17p13.3-ter in sporadic breast cancer is evidence for a putative tumour suppressor gene. *Br J Cancer* **80**: 821–826

Montesinos-Rongen M, Roers A, Küppers R, Rajewsky K and Hansmann ML (1999) Mutation of the p53 gene is not a typical feature of Hodgkin and Reed-Sternberg cells in Hodgkin's disease. *Blood* **94**: 1755–1760

Nagai H, Kinoshita T, Suzuki H, Hatano S, Murate T and Saito H (1999) Identification and mapping of novel tumour suppressor loci on 6p in diffuse large B-cell non-Hodgkin's lymphoma. *Genes Chromosomes Cancer* **25**: 277–283

Osborn RJ and Leech V (1994) Polymerase chain reaction allelotyping of human ovarian cancer. *Br J Cancer* **69**: 429–438

Palzetti D, Crescenzi B, Matteucci C, Falini B, Mertelli MF, van den Berghe H, Mecucci C (1999) Genomic instability and recurrent breakpoints are main cytogenetic findings in Hodgkin's disease. *Hematologica* **84**: 298–305

Sambrock J, Fritsch L and Maniatis E (1989) *Molecular cloning, a laboratory manual* 2nd edition. Cold Spring Harbor Laboratory Press: Cold Spring Harbor

Steichen-Gersdorf E, Baumgartner M, Kreczy A, Maier H and Fink FM (1997) Deletion mapping on chromosome 17p in medulloblastoma. *Br J Cancer* **76**: 1284–1287

 *© 2001 Cancer Research Campaign*

Weber-Matthiesen K, Deerberg J, Poetsch M, Grote W and Schlegelberger B (1995) Numerical chromosome aberration are present within the CD30+ Hodgkin and Reed Sternberg cells in 100% of analysed cases of Hodgkin's disease. *Blood* **86**: 1464–1468

Wolf J, Kapp U, Bohlen H, Kornacker M, Schoch C, Stahl B, Mücke S, von Kalle C, Fonatsch C, Schaefer HE, Hansmann M-L and Diehl V (1996) Peripheral blood mononuclear cells of a patient with advanced Hodgkin's lymphoma give rise to permanently growing Hodgkin-Reed Sternberg cells. *Blood* **87**: 3418–3428

© 2001 Cancer Research Campaign
